# Waste and Want

**Published:** 1857-06

**Authors:** 


### WASTE AND WANT;

### OR, THE PLAGUE OF SERVANTS.

THE rich save, and remain rich; the poor squander, and are poor to their dying day. The rich, especially those who have been so for a generation, or two, or more, are economical from principle, from inclination, from habit; it affords them satisfaction to be so. The conviction within them that it is right, safe, and politic, sustains them in their course.

But there is a class of persons who are of untold injury to society. They are not among the older and more respectable families; their names are recent; their origin nothing to speak of, nor indeed anything to be ashamed of. They are from worthy, industrious parents, who made no pretensions, nor had desired to be considered more than among the common people. By prudent economies, long practised, they have become rich, and having money, at least in perspective, their children have an ever-present, all-absorbing, eating ambition for association with those who are socially above them—if you please the treble upper ten—there by reason of birth, wealth, and merit; and there are a good many such persons in New York. The plan of ascent devised by tnese children of worthy, industrious, and successful parents, and by many who, by chance speculations, have become recently rich, with their vulgar tastes still hanging about them, is through the agency of glare and glitter, and profuse expenditure. They dress in violent colors; their livery is in the extreme of fashion; their dwellings are of brown stone or marble. Swarms of servants loiter about their premises; while milkmen, butchers, green grocers, and all that, rub their hands with glee, at the fortune of getting hold of such liberal customers. Enter their dwellings of an evening, and we feel ourselves in a fairy palace. Gas-lights glitter from gorgeous chandeliers; gilded mirrors dazzle the eyes; the feet tread on the softest velvet; frescoed ceilings compel our admiration; while costly paintings, statues of Italian marble, slabs of beauteous "Sienite," brought from distant Egypt, cover their tables, and delight us with their delicate hues. If these people sail for a season, they "water" at half a dozen places in a single summer, and at each become speedily conspicuous by the remarkable attention shown them by all the servants of the establishment, who were largely "*douceured*" on the first hour of their arrival, and who have a marvellous instinct, as instantaneous as it is certain, in aiding them to find out when they have got hold of a—fool.

The observation of all of us teaches that the end of such persons is speedy and disastrous. They succeed in nothing but in getting clear of their money, in kindling up a flame to enable the community to see them the more clearly go down into their original and more appropriate obscurity. But this is not the last of them. They themselves may be never heard of more; but they leave their slimy trail of pernicious influences behind them, to be felt by worthy people for a life-time. The servants they employed fell into wasteful habits, gormandizing habits, habits of idleness and gossipping. Careless of fuel, careless of food; gas wasted, water wasted, ranges burnt out, crockery broken, silver battered—destruction everywhere the order of the day. Sooner or later these servants get married, and carry with them all these thriftless habits to their own home. In a few months, the hard-working husband—a mechanic, or drayman, or day laborer—opens his eyes to the unwelcome reality, becomes discouraged, idles about, takes to drink, and mutual upbraidings end in quiet desertion or midnight murder. This, we are persuaded, is the large origin of the wife-beatings and wife-murders so often recorded in the morning papers. This accounts for the hordes of neglected children which swarm our streets and besiege the charities of our citizens—for the crowds of apparent widows who drain the treasuries of our benevolent associations in winter time, whose husbands are not dead to the world, but only dead to the women whose thriftless habits were learned in the houses whose masters are in the penitentiaries and whose mistresses have found refuge in some retreat or asylum, if not in some house of infamy.

So much for the servants.

Look for a moment at the influences these servants have in the first families they enter after one of these New York come-downs. Almost the first question asked by Biddy, who has advertised in the *Herald*, is, "What wages do you give? I had eight dollars a month with the last lady—and a nice lady she was, too—but they broke up housekeeping, and have gone to Europe."

"How many have you in family, ma'am? what other help? I suppose you have the washing and baking done out? The fine lady I was with last, made the pastry herself, and we always had as much as we liked."

Then comes the inquisition as to privileges allowed.

1. There is to be an afternoon in the week, to go out and see some cousin, or brother, or other relation—the "afternoon" being from three o'clock until eleven, P. M.

2. Must go to church on Sundays, to return any time before twelve o'clock at night.

3. Privilege to have some other relation to drop in now and then of an evening; which means to have some beau, under the name of brother, come once or twice a week, besides gallanting home on Sunday nights and *their* "evening out," to sit in youı kitchen and enjoy your gas, and fire, and larder, until midnight.

4. Then, there is the occasional privilege. Who ever had a servant that did not have a cousin to die, or a cousin's child to be born, or christened, on an average of two or three times a month? There is an extension of privilege on these "occasional" occasions; Biddy does not expect to return until after breakfast next morning.

The influence which these trifling creatures exercise over weak mistresses, is amazing. They will tell you of the ways and habits of the "grand house I stopped at last," as a means of flattering you into an equal generosity as to high wages, profuse living, and little to do; and, before he knows it, the simple-minded master wakes up to a new idea.

"Husband! Biddy has been living with the * * *, and I am ashamed that anything should be set on the table a second time. It won't do to make turkey hash of the odds and ends of bread left at the several last meals, nor to make a bread pudding of it for the children."

"But, my charming wife! does not cold turkey make a good relish for tea or breakfast, and don't our children all like turkey hash?—why, I like it myself."

"Well! but it looks mean to be so saving and close. The servants will talk about it."

"My dear, delightful lamb, dove, angel, and humming-bird! it would be better to *look* mean, and owe nobody, with some cash on hand always, than to *be* mean, by squandering what we have saved these long years, for the sake of having the good opinion of a parcel of ignorant, thoughtless, thriftless servant girls."

"Husband! that's the way you always talk; but if our children's playmates get to hear that we are penurious and stinting, they will set us down as mean people, and not associate with them. I never thought we would come to this, or I would have married Charles Slasher; he always wanted me, and his father was rich, and left him a large fortune—boo! boo! boo!——"

"Wifey, dear! don't you know that reflections, and upbraidings, and tears, always move me in the opposite direction? Do you know what has became of your old beau, Charley Slasher? One of my Southern correspondents writes me, yesterday, that he is now in the Louisiana Penitentiary, for forgery, with three years to serve. I don't want to follow. Please, say to the girls, their wages are ready, to the end of the present month; and that they are expected to leave after dinner. We will get another set, who will allow me the privilege of dictating in my own house."

Weaker husbands yield to tears and false reasonings; and hence we may see every day the red flag of the auctioneer waving at the door of the aristocrat of an hour, or over the remnant of his "household gods," in the crammed auction rooms in Nassau street, the morning paper stating suggestively, "sold peremptorily, on account of domestic affliction!"

Let all housekeepers who act maturely and from principle, reflect, that in keeping more servants about the house than is necessary for the work to be done—that in giving eight and ten dollars a month to ordinary cooks, nurses, and house girls—that in allowing late hours, and careless handling of crockery, and wastefulness of fuel, and soap, and food, they do but foster habits in their girls which are to be the thorns of their domestic life when they become the heads of families themselves. You do them this wrong without any corresponding benefit to themselves, and without even adding to your own comfort or that of your families; for it is the nature of idleness, waste, and extravagance, to increase in geometrical progression.

If the fanatical humanitarian wants to break the chains of bondage, and let the oppressed go free, let his sympathies and his corresponding personal and pecuniary aids, if he have any, find employment in rescuing our wives from the fear and despotism of the servant girls of this city. Within ten miles of the City Hall there is work enough for them all to do, day and night—a field white to the harvest to-day, and broad enough to give working room to every one of them during the term of their natural lives.

Is there a housekeeping woman in New York who is not in daily "fear," of some sort, of her servants? Rather broad, that question, reader. There is, at least, one family—our own. We have but one fear, and that is—that our "help" will take the underground railroad to Gretna Green, one of these days.

If our girls are not unbearable, we fear to "change," "lest a worse thing come upon us;" and, rather than tempt the untried, we put up, day after day, with a multitude of impositions of one sort or another. We wink at useless waste, and slighted work, and late hours, and loitering conversation at the hall door or front gate, "afraid to speak," lest "offence" should be given. Biddy offends you, deliberately neglecting your directions, wastes your property, consults her ease in preference to your comfort, and yet you live in mortal fear of giving offence; paying her to the last cent of what you agreed to do, and yet she making a systematic experiment as to how little she can do for you without absolute collision.

This plague of servants is a foul spot on our domesticities, and ought to be eradicated. Concerted action among respectable families would effect a happy riddance, as immediate as the total abolition of the Spanish currency, within ten days after the announcement of a passed "act" on the subject. The main points for observance we merely suggest:

1. That no female servant be allowed more than a dollar and a half a week, under any pretense whatever, in private families.

2. That beyond half of each Sunday, all time spent out of the house should be deducted from the weekly wages, in proportion.

3. That receiving visitors be confined to one hour each week, and that hour be from ten to eleven o'clock every Thursday morning.

4. That servants be rigidly required to be in their bed-rooms for the night when the clock strikes ten.

5. That all perquisites be abolished.

6. That nothing is to be given or received, bought or sold, at the front gate, or basement door, except for the master's use.

7. That the basement gate be always kept locked, and opened only for receiving orders for, or delivery of, family supplies.

8. That the washing of clothing and house, and spring cleaning—that all bread-baking and pastry-cooking, be required to be done by the servants in the house; or in case persons prefer baker's bread, and to make their own pastry, then a reasonable deduction be made, for this diminution of requirement, from their weekly wages.

9. That servants be made to understand, that on chance occasions they are to do promptly, willingly, and as a matter of course, whatever may be requested of them, whether it be in the particular line of their engagement or not, on the ground that their time is paid for, and that it is their duty to make themselves generally useful.

10. That all waste and brokerage be justly estimated, and deducted from their weekly wages.

11. That to every courteous and becoming requirement, prompt, and implicit, and quiet obedience be invariably given.

12. That no servant be employed who cannot furnish satisfactory reference from the last employers, which reference is to be authenticated by presentation to the person who gave it.

Here are a dozen regulations, whose rigid enforcement would deliver the wives of New York from an amount of domestic inquietude, and irritation, and worriment, absolutely beyond computation. It would replace discouragement with hope—a heart as heavy as lead with a buoyancy of spirit as light as a feather. It would give sunshine for clouds, smiles for frowns, quietude for storms, and endearments for belligerency.

What else would this revolution accomplish? It would break up habits of idleness, useless waste, and wild extravagance, and foster industry, carefulness, and economy, which would benefit the girls themselves in after life to an untold amount.

In addition, it would sweep away that horde of dirty, ragged boys, girls, and women, who, with basket in hand, go from gate to gate every day the year round, and sometimes thrice a day, for the "leavings" of the table, and which, by reckless and unprincipled servant girls, are always increased by portions of the best in the house—thus encouraging idleness and beggary, and adding daily actual theft to the same.

But then, there are obligations resting on the head of the family likewise, among the most prominent of which are:—

1. Regular weekly pay, in United States coin.

2. A humane consideration for the prejudices, weakness, and ignorance of servants.

3. Special attention to their personal health and comfort, in whatever pertains to these.

4. The avoidance of untimely and unreasonable requisitions.
5. A benevolent forbearance as to occasional short-comings.
6. A kindly and unfamiliar courtesy in expressing every wish, and in enforcing every requirement.

None can ever understand as well as a city practitioner, the wearing, wasting effect which constant domestic irritations have on the tempers, and happiness, and health, of the wife and the mother. Drop by drop will wear away the solid rock; drop by drop will drain the ocean dry; and there is no temper however sweet—no spirit however buoyant—no health however vigorous—but will be soured, and saddened, and broken down, by the incessant annoyances which trifling servants occasion; and every husband, every father, every son, owes it to the wife, the mother, and the sister, who share so large a portion of their affection and their care, to seek the initiation of measures which shall rid us at once of the "*plague of servants.*" It is a worm at the root of family quiet; it eats out a large share of the happiness of domestic life; and we recommend that every husband who does not give this subject an effective consideration, have a mark set upon him, which shall indicate that dissolution of the present marriage tie is his end and aim; and that while the servant plague is doing its fated work unhindered by him, he is unblushingly looking out for a new alliance. Shame on ye, gentlemen!

